# Large-Scale Functional RNAi Screen in *C. elegans* Identifies TGF-β and Notch Signaling Pathways as Modifiers of *CACNA1A*

**DOI:** 10.1177/1759091416637025

**Published:** 2016-03-18

**Authors:** Maria da Conceição Pereira, Sara Morais, Jorge Sequeiros, Isabel Alonso

**Affiliations:** 1 UnIGENe, Institute for Molecular and Cell Biology (IBMC), Instituto de Investigação e Inovação em Saúde (i3S), University of Porto, Portugal; 2 Abel Salazar Institute for the Biomedical Sciences (ICBAS), University of Porto, Portugal; 3 CGPP, Institute for Molecular and Cell Biology (IBMC), Instituto de Investigação e Inovação em Saúde (i3S), University of Porto, Portugal

**Keywords:** RNAi screen, *unc-2*, enhancer/suppressor, disease modifiers

## Abstract

Variants in *CACNA1A* that encodes the pore-forming α_1_-subunit of human voltage-gated Cav2.1 (P/Q-type) Ca^2+^ channels cause several autosomal-dominant neurologic disorders, including familial hemiplegic migraine type 1, episodic ataxia type 2, and spinocerebellar ataxia type 6. To identify modifiers of incoordination in movement disorders, we performed a large-scale functional RNAi screen, using the *Caenorhabditis elegans* strain CB55, which carries a truncating mutation in the *unc-2* gene, the worm ortholog for the human *CACNA1A*. The screen was carried out by the feeding method in 96-well liquid culture format, using the ORFeome v1.1 feeding library, and time-lapse imaging of worms in liquid culture was used to assess changes in thrashing behavior. We looked for genes that, when silenced, either ameliorated the slow and uncoordinated phenotype of *unc-2*, or interacted to produce a more severe phenotype. Of the 350 putative hits from the primary screen, 37 genes consistently showed reproducible results. At least 75% of these are specifically expressed in the *C. elegans* neurons. Functional network analysis and gene ontology revealed overrepresentation of genes involved in development, growth, locomotion, signal transduction, and vesicle-mediated transport. We have expanded the functional network of genes involved in neurodegeneration leading to cerebellar ataxia related to *unc-2*/*CACNA1A*, further confirming the involvement of the transforming growth factor β pathway and adding a novel signaling cascade, the Notch pathway.

## Introduction

Voltage-gated calcium channels are involved in diverse biological processes of excitable cells, such as neurotransmitter release, excitation–contraction, regulation of gene expression, and neuronal migration by coupling electrical activity to intracellular Ca^2+^ signaling. These channels comprise an oligomeric assembly of different subunits that, when altered, were shown to be implicated in several cardiovascular and neurological diseases (Miller, 2001; Mathews et al., 2003).

The human *CACNA1A* gene encodes the α_1_-subunit (pore-forming) of P/Q-type voltage-gated Ca^2+^ channels (Ca_v_2.1). Mutations in this gene have been found to cause a spectrum of neuronal disorders with overlapping phenotypes: episodic ataxia type 2, familial hemiplegic migraine type 1, and spinocerebellar ataxia type 6 (Pietrobon, 2010; Rajakulendran et al., 2012).

*CACNA1A* is evolutionarily conserved, and its ortholog in *Caenorhabditis elegans* (*unc-2*) shares over 60% sequence identity with the human form. In the 1970s, Sydney Brenner’s lab generated the CB55 strain, among others, by Ethyl methanesulfonate (EMS) mutagenesis from the N2 Bristol. The CB55 strain carries the *unc-2* canonical e55 mutant allele, that is, a base-pair substitution that introduces a premature stop codon, rendering the worm to an *unc-2* null phenotype (Mathews et al., 2003; Estevez et al., 2004). This truncating mutation is detected by the translation machinery and its mRNA targeted for nonsense-mediated decay. This culminates in the absence of *unc-2* expression or activity, effectively mimicking a knockout of the *unc-2* gene.

In *C. elegans*, *unc-2* is mainly expressed presynaptically, where its primary role is to mediate fast neurotransmission, although mosaic analysis has also suggested that it can be present both in the pre- and postsynaptic regions – neuronal and muscular functions (Schafer and Kenyon, 1995).

Strains carrying mutations in the *unc-2* gene are mainly characterized by slow and uncoordinated movement, slightly longer body length than wild-type (N2) animals, and constitutive egg-laying defects. The latter is due to their hypersensitivity to serotonin causing *unc-2* mutants to lay eggs with embryos at an abnormally early developmental stage and under inhibitory conditions for the N2 worms. At the molecular level, they show further defects in cholinergic and GABAergic synapses (Schafer and Kenyon, 1995; Mathews et al., 2003).

Overall, behavioral, pharmacological, and molecular phenotypes of the *unc-2* mutant strain closely reproduce the phenotypes associated with vertebrate P/Q-type channels (Rajakulendran et al., 2012). And, even though these genetic defects are rare, their study has already provided insight to common disorders of the central nervous system, such as migraine (Estevez et al., 2004, 2006) and epilepsy (Williams et al., 2004), and may continue further to do so.

To identify modifiers of the uncoordinated phenotype in *unc-2*-related channelopathies, we performed a large-scale functional RNAi screen, using the *C. elegans* strain CB55, which carries a truncating mutation in the *unc-2* gene, and looked for genes that, when silenced, either ameliorated the slow and uncoordinated phenotype of CB55 worms, or interacted to produce a more severe phenotype.

## Materials and Methods

### C. elegans Strains

Nematode strains were handled according to standard methods (Stiernagle, 2006). Strains were grown at 20℃, unless otherwise stated. N2 (Bristol) was used as the wild-type and the CB55 mutant strain, carrying a truncating mutation in the *unc-2* gene, presented the uncoordinated motor phenotype to be targeted by the modifier screen. All strains were provided by the Caenorhabditis Genetics Center, which is funded by the NIH Office of Research Infrastructure Programs (P40 OD010440).

### RNAi Liquid Feeding

The screen was carried out in a 96-well liquid culture format using the *C. elegans* open reading frame (ORF)-RNAi feeding library (Source Bioscience), targeting approximately 11,000 genes, as detailed later.

Bacterial clones from the *C. elegans* ORFeome RNAi library v1.1 (Rual et al., 2004) were grown overnight in Lysogeny broth (LB) culture medium supplemented with ampicillin (100 µg/ml) and tetracycline (12.5 µg/ml) at 37℃ and induced for 1 hr by adding Isopropyl β-D-1 thiogalactopyranoside (IPTG) to a final concentration of 4 mM, prior to incubation with the nematodes. Also, the previous day, hypochlorite egg extraction was performed to obtain synchronized L1 larvae (Stiernagle, 2006). The eggs were left to hatch overnight in the absence of food, at room temperature (20℃ ± 5).

An average of 5 L1-stage worms in M9 Buffer (10μl) were distributed into 96-wells containing 40 μl of RNAi feeding bacteria, suspended in Nematode growth medium (NGM) media supplemented with ampicillin, tetracycline, and IPTG (Lehner et al., 2006). Feeding plates were afterward incubated at 20℃ for 7 days with shaking. The H115 strain carrying the empty vector pL4440 and the *hil-5* ORF (previously reported to have no effect in the worm phenotype; Fraser et al., 2000; Rual et al., 2004; Sonnichsen et al., 2005) were used to control bacterial feeding and the standalone induction of the RNAi machinery. The *lpin-1* ORF, producing a severe developmental phenotype (Rual et al., 2004; Sonnichsen et al., 2005), was used to control RNAi induction efficiency in every 96-well plate.

Time-lapse imaging of worms in liquid culture was used to assess changes in thrashing behavior of the *C. elegans* strains. Every well was recorded for 30 s at 10 Hz, using an automated recording stage coupled to a charge-coupled device-equipped stereoscope. A single thrash was deﬁned as a complete change in the direction of the body down the midline.

Raw imaging data were analyzed with open source wrMTrck plugin for Image J, and the thrashing results were loaded on CellHTS2 for further analysis (Pelz et al., 2010). One important aspect of data acquisition and analysis regards full automation, which allowed the reduction of random errors and bias introduced by the experimenter.

A primary screen was carried out on the CB55 mutant strain with duplicates and, after analysis, RNAi hits were considered when separated from the mean at least two standard deviations. Genes fulfilling these criteria were filtered for severe phenotypes, such as Emb and Ste, already reported in WormBase (WS235; Chen et al., 2005); these were excluded from further analysis. The remaining clones were taken into a second and third assay rounds, using the approach described earlier, with the exception that both the N2 and CB55 strains were used in parallel and experiments were run in triplicates. This confirmation assays had the purpose to fulfil two goals: first, to confirm reproducibility of the effect observed in the primary screen; and, second, to identify genetic interactions specific of the CB55 mutant.

All final clone hits were directly sequenced to verify their identity using the M13 universal primer sequence.

### Functional Gene Networks

The top 40 genes related to the ones identified in the genetic screen were plotted using Cytoscape v3.3.1 (Shannon et al., 2003) and GeneMANIA App (Montojo et al., 2010), which allowed for network generation, according to Gene ontology (GO) term enrichment analysis and gene function prediction (only terms with Q value ≤ 10^−4^ were considered).

## Results and Discussion

The aim of our study was to gather information on the genes and pathways that may act as modifiers in diseases associated to the *CACNA1A* gene. To accomplish this, we performed a genetic screen in the uncoordinated CB55 *C. elegans* strain, followed by ontology analysis to identify the main biological processes that acted as suppressors or enhancers of this mutant phenotype.

### Screen Design

Because the *CACNA1A* gene is physiologically related to an expanding spectrum of human neurological disorders that implicate transient or progressive motor deficits, we performed a genetic screening for suppressors and enhancers of the motor phenotype in a model system. In *C. elegans*, the *CACNA1A* ortholog, *unc-2*, is highly homologous to its human counterpart and, when mutated, causes uncoordinated body movement, among other features that greatly reproduce the phenotype associated with human disease.

We took the CB55 strain and performed a large-scale functional RNAi screen in liquid culture, using the ORFeome library (Lamesch et al., 2004), targeting approximately 11,000 *C. elegans* genes.

Due to the mutant’s hyperactive egg-laying behavior, the average number of worms distributed per well had to be reduced to the minimum number that guarantied no empty wells.

**Figure 1.**
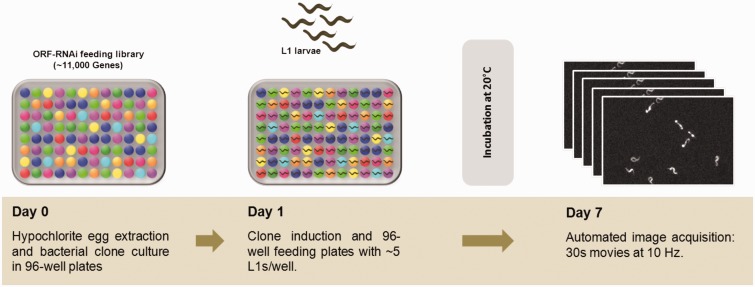
RNAi screen workflow. On the first day (Day 0), a 96-well plate was inoculated with the RNAi bacterial clones, and the CB55 eggs are extracted by bleaching gravid worms. The larvae then hatched overnight, and in the absence of food, they arrest at the L1-stage. Next day (Day 1), after induction of RNAi expression, each bacterial clone is fed to approximately five synchronous worms, which during the period of incubation grow and lay eggs. On Day 7, the progeny has hatched, and each well is imaged for 30 s to record their movement. ORF = open reading frame.

Another concern, partially related to the egg-laying behavior of this strain, is the development rate. While for the N2 wild-type strain one can observe and score progeny after 4 days of feeding (at 20℃), the CB55 worms need more time to grow and reach adulthood (probably due to slower pharyngeal pumping and, therefore, poor feeding), and also, as the eggs are prematurely laid, they take significantly longer to hatch. All these reasons account for the 7 days of feeding we found to be required prior to progeny imaging and scoring of thrashing behavior. Figure 1 shows an overview of the screen workflow and conditions.

### *unc-2* Enhancer/Suppressor Large-Scale RNAi Screen

#### Primary screen

A primary screen was carried out on the CB55 mutant strain with duplicates, and, after image acquisition and thrash analysis, the raw data were further explored through CellHTS2. Because the worm nervous system is known to be somewhat refractory to systemic RNAi, only the progeny was considered during the analysis, as they had been exposed to partial gene depletion since embryogenesis.

**Figure 2.**
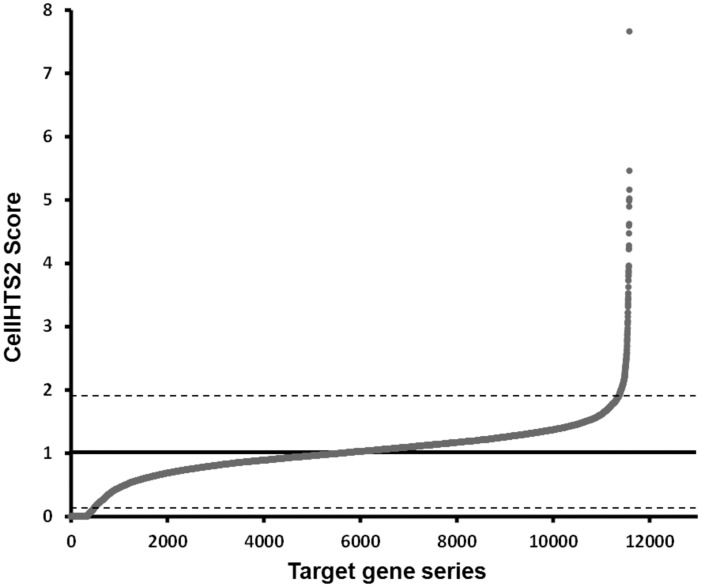
Graphical representation of the CellHTS2 score results series of the primary screen. Data analysis using CellHTS2 revealed a mean score distribution around 1.005 ± 0.438, that is, ≥ 95% of the RNAi tested had no effect over the CB55 phenotype. Genes were considered preliminary RNAi hits, when scoring at least two standard deviations away from the mean (highlighted by the dashed line). At this stage, the screen yielded 676 putative RNAi hits.

Data analysis revealed a mean score distribution around 1.005 ± 0.438, meaning that approximately 95% of the RNAi tested had no effect over the CB55 phenotype. We considered as preliminary RNAi hits, those genes with scores at least two standard deviations from the mean. Figure 2 shows a graphical representation of the CellHTS2 score results series, highlighting those outside the mean ± 2*SD*. The primary screen yielded 676 putative RNAi hits. Among these, however, were putative enhancers, producing severe phenotypes that have been previously reported in WormBase (WS235; Chen et al., 2005) for the N2 wild-type strain. Several RNAi already reported to produce embryonic lethality (Emb) and adult sterility (Ste) were excluded from further analysis, which reduced the number of potential hits to 350, for further confirmation.

#### Validation screens

The remaining bacterial clones from the primary screen (350) were taken into two consecutive assays using the same approach with the exception that both the N2 and CB55 strains were used in parallel and experiments were run in triplicates. This allowed us to confirm reproducibility of the phenotype observed in the primary screen, and, by introducing the N2 wild-type strain, we were able to discriminate between genetic interactions specific of the *unc-2* mutant and those common to both strains, as well as discordant effects (Table 1). Overall, bacterial clones targeting 37 genes consistently showed reproducible results. Groups II to VI include discordant RNAi effects between N2 and CB55, while group VII comprises a list of genes that when downregulated increase motor ability in both strains, having a more generic effect. Furthermore, taking into account the available expression data in WormBase (WS242; Chen et al., 2005), at least 75% of the genes identified are known to be expressed in the *C. elegans* neurons.

**Table 1.** Genetic Enhancers and Suppressors of the *unc-2* Motor Phenotype.

Group	Gene	Thrashing rate (score)		Expression	Protein
		N2 (3.3)	CB55 (0.7)		
I	*ncbp-1*	− (0.0)	− (0.6)	N/A	NCBP-1 – Nuclear Cap-Binding Protein, orthologous to human NCBP1
II	*vps-45*	= (4.0)	− (0.0)	Nervous system	VPS-45 – Related to yeast Vacuolar Protein Sorting factor
	M57.2	= (3.7)	− (0.0)	N/A	N/A
	*snr-5*	= (4.1)	− (0.5)	Nervous system	SNR-5 – Small Nuclear Ribonucleoprotein
III	*cdc-6*	+ (5.1)	− (0.2)	Seam cell	CDC-6 – Cell Division Cycle related
	*xpf-1*	+ (3.4)	− (0.4)	Gonad	XPF-1 – (Xeroderma Pigmentosum group F) DNA repair gene homolog
	*npax-1*	+ (4.2)	− (0.2)	Head neurons	NPAX-1 – N-terminal PAX (PAI domain only) protein
	*sago-2*	+ (4.9)	− (0.2)	N/A	SAGO-2 – Synthetic secondary siRNA-deficient ArGOnaute mutant
	*top-1*	+ (4.6)	− (0.1)	Nervous system	TOP-1 – TOPoisomerase
	*klp-3*	+ (4.2)	− (0.0)	Marginal cell	KLP-3 – Kinesin-Like Protein
	*dbl-1*	+ (4.1)	− (0.1)	Nervous system	DBL-1 – DPP/BMP-Like
	*sec-8*	+ (6.2)	− (0.0)	N/A	SEC-8 – yeast SEC homolog
	Y47G6A.19	+ (4.4)	− (0.1)	N/A	Y47G6A.19 – orthologous to human carboxypeptidase A and B
	*ppfr-4*	+ (5.6)	− (0.1)	N/A	PPFR-4 – Protein Phosphatase Four Regulatory subunit
	*aph-2*	+ (4.4)	− (0.2)	Oocytes	APH-2 – ortholog of nicastrin
IV	*unc-97*	− (0.0)	= (0.6)	Nervous system	UNC-97 – LIM domain-containing protein of the PINCH family
V	*egl-43*	− (1.4)	+ (1.4)	Head neurons	EGL-43 – Zinc finger protein
	*ceh-21*	− (1.1)	+ (1.8)	Nervous system	CEH-21 –*Caenorhabditis elegans* Homeobox
VI	*daf-7*	= (3.5)	+ (0.9)	ASI neurons	TGF-β
	*tbx-38*	= (3.2)	+ (1.3)	Head neurons	TBX-38 – T box transcription factor
	*prx-5*	= (3.1)	+ (1.4)	Nervous system	PRX-5 – ortholog of the human receptor for type I peroxisomal targeting signal protein
	K10C3.5	= (3.1)	+ (2.2)	N/A	K10C3.5 – ortholog of budding yeast Ria1p/Efl1p and human EFTUD1
	*unc-43*	= (3.2)	+ (0.9)	Nervous system	UNC-43 – ortholog of type II calcium/calmodulin-dependent protein kinase (CaMKII)
	*pat-12*	= (3.3)	+ (101)	Reproductive system, hypodermis, rectal epithelium	PAT-12 – Paralysed Arrest at Twofold
	*sedl-1*	= (3.4)	+ (0.9)	N/A	SEDL-1 – human SEDL (spondyloepiphyseal dysplasia tarda) related
	Y54E10A.10	= (3.1)	+ (1.7)	N/A	N/A
	*unc-51*	= (3.1)	+ (1.0)	Nervous system	UNC-51 – serine/threonine protein kinase orthologous to *Saccharomyces cerevisiae* autophagy protein Atg1p and the vertebrate ULK proteins
	*sma-2*	= (3.4)	+ (2.2)	Head neurons	SMA-2 – orthologous to Smad1
VII	*flp-4*	+ (4.6)	+ (1.3)	Nerve ring	FLP-4 – FMRF-Like Peptide
	*nhr*-74	+ (4.9)	+ (0.9)	Seam cell	NHR-74 – Nuclear Hormone Receptor family
	*sma-6*	+ (4.9)	+ (1.0)	ASI neurons	SMA-6 – serine/threonine protein kinase orthologous to type I TGF-β receptor
	*daf-14*	+ (4.0)	+ (0.9)	Nerve ring, AVJ, RIV	DAF-14 – orthologous to Smad2
	*nkcc*-1	+ (7.1)	+ (0.9)	N/A	nkcc-1 – Na-K-Cl Cotransporter homolog
	R107.5	+ (5.5)	+ (.09)	N/A	N/A
	*ntr*-1	+ (5.0)	+ (1.5)	ASEL, ADF, BDU, ASH, PQR	NTR-1 – NemaTocin Receptor, orthologous to the human vasopressin receptor type 2
	*pqn*-85	+ (4.1)	+ (0.9)	N/A	PQN-85 – Prion-like-(Q/N-rich)- domain-bearing protein, orthologous to human NIPBL
	Y51H1A.3	+ (4.1)	+ (0.9)	N/A	Y51H1A.3 – orthologous to human NDUFB8

*Note*. Motor function: (+) improved, (=) no change, and (−) worsened; N/A = information not available; TGF = Transforming growth factor β: DPP/BMP=Bone morphogenic protein.

### Reduced Notch Signaling Aggravates CB55 Incoordination

**Figure 3.**
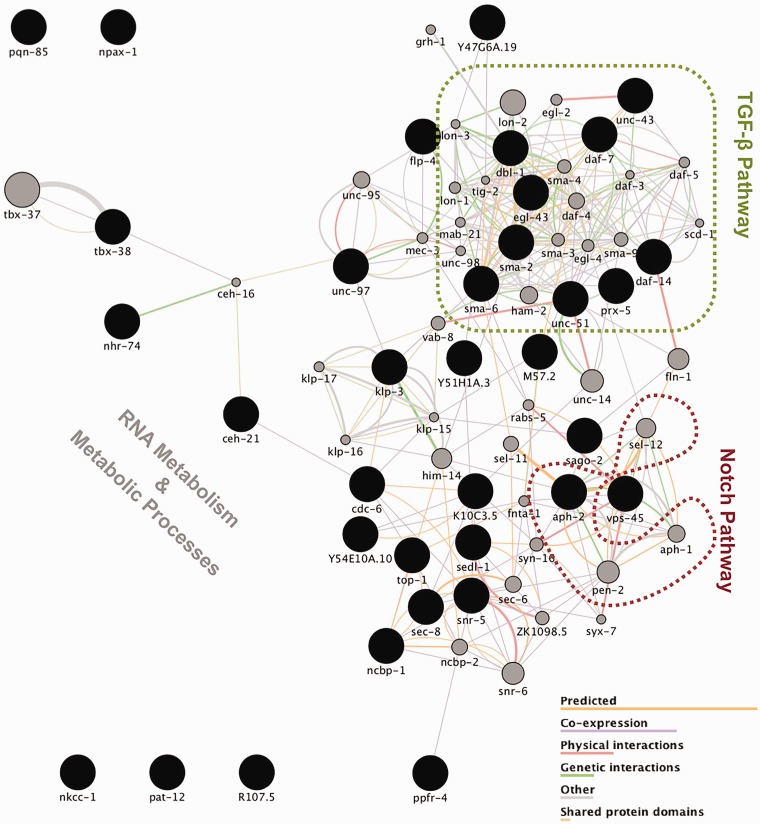
Gene network of enhancer and suppressor genes of the *unc-2* phenotype. The top 40 genes related to those 37 identified in the genetic screen were plotted using Cytoscape v3.3.1 and GeneMANIA App, which allowed for the network generation according to GO term enrichment analysis and gene function prediction. TGF-β and Notch signaling cascades were the main pathways to be identified by the genetic screen as involved in modulating *unc-2* lethargy. Black-colored circles depict direct hits from the screen, while gray-colored ones represent the 40 genes most related to hits, sized according to their weight in the network. The significance of the involvement of other genes relating to RNA metabolism and basic cellular metabolic processes, in light of the available data, remains elusive. TGF-β = transforming growth factor β.

The *unc-2* genetic interaction screen allowed the identification of several genes that, when downregulated, have an effect on the CB55 strain’s uncoordinated movement. Further functional network analysis, using Cytoscape (Figure 3), allowed us to reduce the complexity of the obtained data and pinpoint individual and interconnected pathways with a major impact on phenotype. Among these is the Notch signaling pathway. We have found that *aph-2* silencing results in an aggravation of *unc-2* uncoordinated phenotype. This gene is the worm ortholog of nicastrin, one of the proteins that constitute γ-secretase, a multimeric protease composed of presenilin, nicastrin, Aph1, and Pen2 (Crump et al., 2013); γ-secretase cleaves the amyloid precursor protein to release the Aβ peptide known to be involved in Alzheimer’s disease pathogenesis. In addition, this protease also cleaves Notch, a key step of the Notch signaling pathway, which plays an essential role not only in cell fate decisions during development but also in the adult brain, including maintenance and differentiation of neuronal stem cells, structural and synaptic plasticity, as well as neuronal survival (Ables et al., 2011). To date, no enhancers for the *unc-2* phenotype are known. From the genes belonging to the Notch pathway, *lin-12* (Notch) and *sel-12* (PEN-1/2) are not available in the RNAi library, *pen-2* (PEN2) knockout levels showed no effect on CB55, *aph-2* (Nicastrin) consistently worsened the phenotype, and *aph-1* (APH1), going back to the initial screen, scored on top of the cutoff for worsening the phenotype and thus did not carry on for further analysis, nevertheless showing a clear trend for half of the gamma secretase complex.

Table 1 highlights a phenomenon very common in many neurological disorders—context dependency—as discordant effects on N2 and CB55 stains arise from knockdown with the same RNAi. The phenotypic outcome is dependent on the presence or absence of a functional *unc-2* channel.

Our results suggest that downregulation of nicastrin, and perhaps APH1, may result in decreased Notch cleavage and consequent impairment of Notch-dependent transcriptional activation, aggravating the *unc-2* motor phenotype. Consistent with our findings, the important role of this pathway in cerebellar ataxia is further strengthened by the fact that other Notch signaling components (RBP-J/Su(H)) have been identified as modulators of pathogenesis in a *Drosophila* SCA17 model (Ren et al., 2011). Moreover, ataxin-1, the protein mutated in SCA1, and its related protein BOAT1 have also been found to be components of the Notch signaling pathway (Tong et al., 2011).

### TGFβ and CaMKII Signaling Downregulation Partially Rescues the *unc-2* Phenotype

The gene network plotted in Figure 3 clearly evidences the importance of the TFG- β pathway for the *unc-2* genetic background. We found that downregulation of several genes involved in this signaling pathway partially rescue the *unc-2* uncoordinated phenotype. Among these are *daf-7*, which encodes the worm ortholog for the transforming growth factor β (TGF-β) superfamily; *sma-2*, a receptor-regulated Smad protein (R-Smad), orthologous to Smad1; *sma-6*, the ortholog for the type I TGF-β receptor; and *daf-14* the worm ortholog for Smad2. In fact, partial knockdown of *sma-6* and *daf-14* not only improved CB55 motor function but also had a positive impact in the motor ability of the wild-type strain, as evidenced in Table 1.

The TGF-β is a pleiotropic cytokine that regulates a diverse range of cellular processes, such as proliferation, differentiation, migration, and apoptosis. In humans, the various TGF-β isoforms are expressed in neurons, and glial cells and its receptors are found throughout the central nervous system (Shi and Massague, 2003). TGF-β response is mediated by a high-affinity transmembrane receptor serine/threonine kinase complex, consisting of the TGF-β type I (TRI) and type II (TRII) receptors. Ligand interaction with a TRII dimer recruits and activates TRI, which in turn phosphorylates the receptor-regulated Smad proteins (Smad2 and 3), which are then translocated into the nucleus where they regulate transcription of their target genes (Patterson and Padgett, 2000; Shi and Massague, 2003). This process is well conserved in *C. elegans* (Patterson and Padgett, 2000).

Our results reinforce the previous association of the TGF-β pathway to the lethargic phenotype of the CB55 strain (Estevez et al., 2004). In their study, Estevez et al. crossed null strains for various components of the worm TGF-β pathway to find that ablation, one-by-one, of most of these genes was able to partially rescue *unc-2* lethargy to different degrees. In our screen, only some of these genes were shown to change the phenotype, what can be readily accounted by partial depletion versus null alleles; however, they were fully sufficient to identify the entire pathway through network analysis.

The TGF-β family of cytokines comprises two subfamilies: the TGF-β/Activin/Nodal and the bone morphogenetic protein/growth and differentiation factor/Müllerian-inhibiting substance subfamily (Shi and Massague, 2003). Interestingly, both in a previous study (Estevez et al., 2004) and in this screen, the *dbl-1* gene, which encodes a bone morphogenetic protein-like protein that binds the TGF-β receptor, has either shown no effect, or in our case, to enhance motor deficits, raising the possibility that, in the *unc-2* background, the signaling pathway dissociates based on the ligand subfamily initiating the cascade to produce a different response.

Unc-2-dependent Ca^2+^ influx has long been hypothesized to be linked to *unc-43*/CaMKII regulation, impacting gene expression symmetry in the neurons (Troemel et al., 1999), neuronal migration (Tam et al., 2000), and tyrosine hydroxylase (*tph-1*) upregulation (Estevez et al., 2004). Here, we show that partial depletion of *unc-43* has no effect over N2 coordination; however, in the *unc-2* null background, it partially rescues lethargy, confirming its role in the *unc-2* signaling cascade. Although the combination of these two null alleles has failed to clearly establish this connection, because the outcome was enhanced lethargy, one could argue that the null allele might not be the best suitable model for a protein of critical importance in many cellular pathways, such as *unc-43*. In fact, it has been shown that different levels of downregulation are more sensitive in terms of detecting genetic interactions among essential genes (Fievet et al., 2013).

Apart from *dbl-1*, all other members of the TGF-β cascade align perfectly with the expected partial suppression of the phenotype (*daf-7*, *daf-14*, *sma-2*, and *sma-6*). Looking back into the primary screen, *daf-8* and *sma-3* scored right on the cut-off line for improving the phenotype, and although no further testing was performed, it only supports the overall trend: partial suppression of the *unc-2* phenotype. Even *unc-43* that in previous studies was shown to have unexpected enhanced lethargy, partially improved CB55’s motility in our study, most likely due to partial knockdown instead of full knockout.

**Figure 4.**
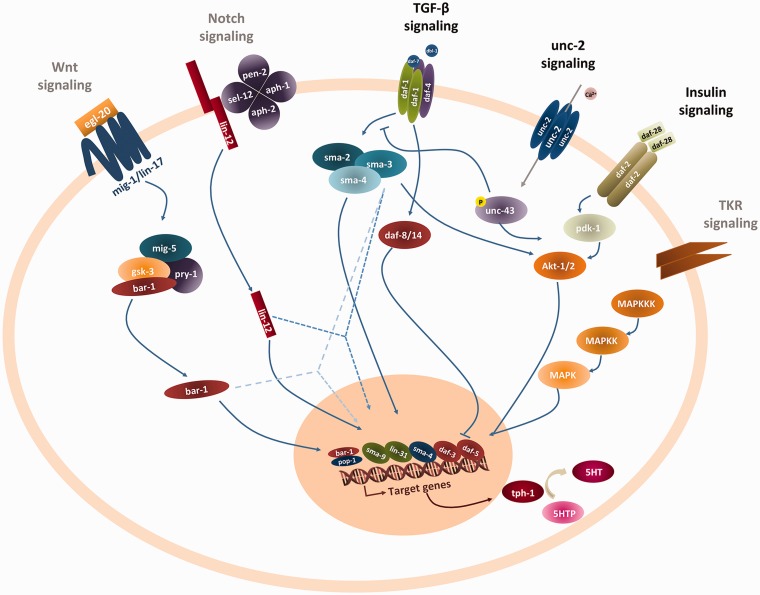
Crosstalk between *unc-2* signaling and other cell signaling pathways. Key intracellular mediators of the *unc-2*, insulin, TGF-β, Notch, TRK, and Wnt pathways are depicted. Pathways identified by black heading are already known modifiers of the *unc-2* phenotype, the remaining, colored gray, depict parallel pathways that, at a given point down the cascade, interact with the already established *unc-2*, insulin, or TGF-β pathways (dashed lines). The unc-2 null allele (CB55) modifier screens support that the majority of genetic modifiers (enhancers or suppressors) are more likely in different parallel pathways like insulin and TGF-β. The figure shows other major cell signaling mechanisms that crosstalk with insulin and TGF-β and may be a source of more *unc-2* modifiers. TGF-β = transforming growth factor β.

The only gene identified that is directly downstream of *unc-2* signaling is *unc-43* (CaMKII; Figure 4). The other genes, including those belonging to the previously associated TGF-β pathway, are in parallel signaling cascades to *unc-2*. Figure 4 depicts other major cell signaling mechanisms that crosstalk with insulin and TGF-β (previously established to interact with the *unc-2* cascade; Estevez et al., 2006; Guo and Wang, 2009; Andersson et al., 2011) such as the Wnt and TKR pathways that may very well be a source of more *unc-2* modifiers. Our library covered only half the Notch pathway genes, and a small minority of genes involved in Wnt and TRK signaling, thus the question on their possible involvement remains.

We have expanded the functional network of genes related to *unc-2* functional role in disease, from the previously reported serotonergic and insulin signaling (Estevez et al., 2006), further confirming the involvement of the TGF-β pathway and adding the Notch pathway as a novel signaling cascade.

The fact that all *unc-2* suppressor genes found to date only partially rescue the phenotype argues in favor of a multifactorial network of modifiers that greatly reflect the phenotypic variability of patients carrying *CACNA1A* mutations, with presentation of symptoms in diverse combinations.

## Conclusions

Our results provide new insight into the genes and pathways that may contribute to neuronal dysfunction and death in calcium channel-related cerebellar ataxia, with an emphasis in specific cellular pathways, particularly TGF-β and Notch signaling. Different approaches have been used to search for modifiers and deregulated pathways in neurodegenerative disorders. We have combined a large-scale genetic screen in *C. elegans*, with functional network analysis, to identify genes and pathways important for cerebellar ataxia-related neurodegeneration that may constitute candidate targets for therapeutical interventions.
